# OTalign: optimal transport alignment for remote protein homologs using protein language model embeddings

**DOI:** 10.1093/bioinformatics/btag476

**Published:** 2026-06-30

**Authors:** Minsoo Kim, Hanjin Bae, Gyeongpil Jo, Kunwoo Kim, Jejoong Yoo, Keehyoung Joo

**Affiliations:** Department of Physics, Sungkyunkwan University, Suwon 16419, Korea; Department of Physics, Sungkyunkwan University, Suwon 16419, Korea; Department of Physics, Sungkyunkwan University, Suwon 16419, Korea; Department of Physics, Sungkyunkwan University, Suwon 16419, Korea; School of Computational Sciences, Korea Institute for Advanced Study, Seoul 02455, Korea; Center for Advanced Computation, Korea Institute for Advanced Study, Seoul 02455, Korea

## Abstract

**Motivation:**

Protein sequence alignment is a crucial task in bioinformatics, yet aligning remote homologs with low sequence identity remains a longstanding challenge, particularly due to the difficulty of handling gaps. We introduce a new method that applies Optimal Transport (OT) theory to sequence alignment, providing a mathematically principled framework for modeling residue matches and gaps.

**Results:**

OTalign formulates sequence alignment as an entropy-regularized unbalanced optimal transport (UOT) problem over embeddings derived from protein language models (PLMs). Unlike traditional methods, it introduces position-specific gap penalties that adapt to each sequence pair. On challenging remote-homolog benchmarks (SABmark, MALIDUP, MALISAM), OTalign consistently outperforms baselines (Needleman-Wunsch, HHalign) and recent PLM-based methods (PLMAlign, DeepBLAST), achieving F1 scores of 0.594 on SABmark Superfamily and 0.358 on SABmark Twilight. Furthermore, OTalign provides a quantitative and interpretable metric of how effectively PLM embeddings represent sequence similarity relationships. Finally, its differentiable nature enables end-to-end fine-tuning of PLMs, establishing a framework for learning embeddings explicitly optimized for alignment tasks.

**Availability and implementation:**

This code is available at https://github.com/DeepFoldProtein/OTalign.

## 1 Introduction

Protein sequence alignment has long served as a cornerstone of bioinformatics, enabling the construction of multiple sequence alignment (MSA) and supporting structure-based prediction pipelines (Needleman and Wunsch 1970, Smith and Waterman 1981, Henikoff and Henikoff 1992, Thompson *et al*. 1994). In modern AI-based protein structure prediction methods such as AlphaFold 2 (Jumper *et al*. 2021), accurate alignment remains indispensable, as it underpins both MSA construction and structural template utilization. However, alignment performance declines significantly in the so-called “twilight zone” (10–25% sequence identity), where traditional similarity measures fail to detect remote homologs (Rost 1999). Addressing this regime is essential for uncovering distant evolutionary relationships and exploring the diverse fold space of proteins.

Recent advances in protein language models (PLMs) such as ESM-2, Ankh, and ProtT5 have introduced a powerful paradigm for learning sequence-based representations that capture structural and evolutionary features (Rao *et al*. 2020, Rives *et al*. 2021, Elnaggar *et al*. 2022, 2023, Lin *et al*. 2023, Chen *et al*. 2025). Although PLMs were originally developed to bridge the gap between sequence and structure, with a primary focus on improving protein structure prediction, these developments have motivated a variety of approaches that adapt PLM embeddings for alignment and homology search, such as PLMAlign (Liu *et al*. 2024). DEDAL learns residue-level substitution scores and position-dependent gap costs and performs alignment with dynamic programming (DP), including differentiable DP during training (Llinares-López *et al*. 2023). Other works, such as DeepBLAST/TM-Vec (Hamamsy *et al*. 2024) and differentiable Smith–Waterman/MSA (Petti *et al*. 2023), used soft or differentiable DP formulations to learn or score alignments. In addition, PLM-informed HMM approaches (e.g. learnMSA2 (Becker and Stanke 2024)) optimize over a distribution of alignments and can incorporate position-specific indel behavior.

In this work, we introduce *OTalign*, which formulates protein sequence alignment as an entropy-regularized *unbalanced optimal transport* (UOT) problem defined over PLM-derived embeddings (Cuturi 2013, Chizat *et al*. 2018, Pham *et al*. 2020). While classical optimal transport provides a principled framework for comparing distributions, we extend it to UOT by relaxing the mass conservation constraint. In this reformulation, transporting mass between residues corresponds directly to establishing matches in the alignment, whereas insertions and deletions naturally emerge as mass creation or destruction events. This perspective makes UOT particularly well-suited for handling biological gaps (insertions/deletions), providing a principled alternative to fixed affine gap penalties. The solving process via the Sinkhorn-Knopp algorithm (Cuturi 2013, Pham *et al*. 2020) ensures computational efficiency with O(NM) complexity and is highly optimized for GPU-based parallelization. Furthermore, the fully differentiable nature of our formulation enables the end-to-end fine-tuning of PLMs for alignment tasks.

Our main contributions are as follows: (1) A new alignment framework: OTalign formulates protein sequence alignment as a UOT problem, achieving state-of-the-art performance on remote homolog benchmarks through UOT-based parameterization. (2) A quantitative probing tool for PLM embeddings: OTalign quantitatively evaluates how well PLM model embeddings capture sequence-structure relationships across diverse PLMs (e.g. ESM-1b and ESM-2 (Rives *et al*. 2021, Lin *et al*. 2023), ProtT5 (Elnaggar *et al*. 2022), Ankh and Ankh3 (Elnaggar *et al*. 2023, Alsamkary *et al*. 2025)). (3) Fine-tuning capability: The differentiable nature of OTalign enables end-to-end fine-tuning with reference alignments, consistently improving both precision and recall. (4) Downstream validation: We demonstrate that OTalign template realignment improves AlphaFold 2 structure prediction on CASP15 domains, particularly for TBM-hard targets where remote homolog templates are available but poorly aligned by standard methods. Together, these contributions establish OTalign as both a high-performance alignment tool and an interpretable framework for analyzing and improving PLM representations.

## 2 Materials and methods

Optimal Transport (OT) provides a mathematical framework for finding the most efficient way to transform one distribution of mass into another. Conceptually, it is akin to calculating the minimal effort required to move a pile of sand into a desired shape. In sequence alignment, this translates to finding the lowest-cost matching residues between two sequences, where the cost reflects their dissimilarity. However, standard (or “balanced”) OT enforces strict mass conservation, requiring that mass is fully transported under fixed marginals, which leaves no principled way to represent unmatched residues (gaps). This constraint is ill-suited for biological sequences, whose lengths often differ due to frequent insertions and deletions (indels).

### 2.1 Unbalanced optimal transport formulation of sequence alignment

To address this, we formulate sequence alignment using *unbalanced* optimal transport (UOT), which provides the necessary flexibility to model indels. UOT relaxes the strict mass conservation constraint by allowing mass to be created or destroyed at a certain penalty cost. In our framework, this elegantly corresponds to opening gaps: unmatched residues in either sequence are treated as “destroyed” or “created” mass. This provides a principled mechanism for handling gaps without relying on traditional hand-tuned affine penalties.

Given two protein sequences of lengths *M* and *N*, a PLM with embedding dimensionality *D* produces per-residue embeddings $x\in R^{M\times D}$ and $y\in R^{N\times D}$. We then define a cost matrix $C_{ij}=1-\text{cos}({\hat{x}}_{i},{\hat{y}}_{j})$, where ${\hat{x}}_{i}$ and ${\hat{y}}_{j}$ denote normalized residue embeddings. The optimal transport plan $\Gamma \in R_{\geq 0}^{M\times N}$ is obtained by minimizing the entropy-regularized UOT objective:


(1) $$\begin{array}{c} \Gamma^{*}=\arg\;\min_{\Gamma }\;\;\langle \Gamma ,C\rangle +\varepsilon \;\text{KL}\left(\Gamma \;\|\;ab^{⊤}\right)\\ +\tau \left[\text{KL}\left(\Gamma 1\;\|\;a\right)+\text{KL}\left(\Gamma^{⊤}1\;\|\;b\right)\right] \end{array}$$

where $\langle \Gamma ,C\rangle =\sum_{ij}\Gamma_{ij}C_{ij}$. a and b are uniform marginal distributions. ε>0 and τ>0 denote the entropy-regularization and marginal relaxation parameters, controlling the smoothness of the transport plan and the tolerance to indels, respectively. 1 denotes an all-ones column vector of appropriate dimension, such that Γ1 and $\Gamma^{⊤}1$ represent the row and column sums of Γ, respectively.

The objective $\Gamma^{*}$ can be solved efficiently using the Sinkhorn-Knopp algorithm (Cuturi 2013, Feydy *et al*. 2018, Pham *et al*. 2020). In practice, each Sinkhorn iteration performs dense matrix-vector scaling over the Gibbs kernel, with a computational complexity of O(MN) per iteration. It operates by alternately normalizing the rows and columns of a kernel matrix derived from the cost matrix *C* and regularization parameter ε. This process can be visualized as repeatedly adjusting the “flow” of mass between the two sequences until the desired marginal constraints (or their relaxed UOT equivalents) are satisfied. A key advantage is its implementation through simple, highly parallelizable matrix-vector operations, making it computationally fast and well-suited for modern hardware like GPUs. Furthermore, the entropy regularization ensures the solution is unique and differentiable, a critical property that enables our end-to-end fine-tuning framework.

### 2.2 Deriving alignment parameters from the transport plan

The raw output of the UOT solver is a probabilistic transport plan $\Gamma \in R_{\geq 0}^{M\times N}$. We interpret Γ as a soft alignment map and derive position-specific scores and gap penalties for standard dynamic programming.

#### 2.2.1 Deriving a position-specific match score matrix

The transport plan Γ is interpreted as a soft joint probability distribution. From the normalized probability distribution, $\gamma_{ij}=\Gamma_{ij}/\sum_{i^{\prime },j^{\prime }}\Gamma_{i^{\prime }j^{\prime }}$, we compute a match score matrix $S\in R^{M\times N}$ based on Pointwise Mutual Information (PMI):


(2) $$S_{ij}=\alpha \cdot \;\text{log}\;\left(\frac{\gamma_{ij}}{\gamma_{i}\gamma_{j}}\right)$$

where $\gamma_{i}=\sum_{j^{\prime }}\gamma_{ij^{\prime }}$ and $\gamma_{j}=\sum_{i^{\prime }}\gamma_{i^{\prime }j}$ are the marginal probabilities. PMI quantifies the co-occurrence probability of residues relative to expectation, identifying residue pairs that are statistically more likely to align—often reflecting conserved functional or structural roles. The hyperparameter α (score_scale) controls the scale of match scores relative to gap penalties.

#### 2.2.2 Deriving position-specific gap penalties from dual potentials

Unlike traditional fixed-cost models, OTalign introduces dynamic, position-specific gap penalties. Crucially, our ablation study (Table 2) reveals that the performance gain comes almost exclusively from the UOT *dual potentials*, $f\in R^{M}$ and $g\in R^{N}$. These dual potentials inherently quantify the cost of leaving a residue unmatched in the optimal transport plan, making them a principled proxy for alignment gaps. This allows OTalign to penalize gaps more heavily in conserved regions while remaining flexible in variable loops, adapting to the global context of the alignment. The detailed transformation from dual potentials to gap penalties is described in the Supplementary Section S4, available as supplementary data at *Bioinformatics* online.

#### 2.2.3 Final alignment with dynamic programming

Once the position-specific match score matrix *S* and gap penalties are obtained, OTalign performs sequence alignment using a standard Needleman-Wunsch dynamic programming (DP) decoder. In this step, the probabilistic transport plan Γ provides a soft alignment map that guides the DP algorithm, ensuring that the final alignment path follows regions of high transport probability while respecting the gap penalties. This hybrid decoding, which combines continuous UOT optimization with discrete DP alignment, yields a stable, interpretable alignment consistent with both the probabilistic transport structure and biological constraints.

### 2.3 Supervised fine-tuning

We fine-tuned the ESM-1b model by minimizing the Kullback-Leibler (KL) divergence between the predicted transport plan and a ground-truth plan derived from structural alignments. This procedure trains the PLM to generate embeddings that are better suited for alignment.

The Sinkhorn algorithm makes our UOT formulation well-suited for the fine-tuning because it is fully differentiable with respect to the input embeddings. The entropy regularization term plays a crucial role in the training, as it makes the UOT objective strictly convex, guaranteeing a unique and smooth solution and stable differentiation with respect to the input embeddings. See Supplementary Section S1, available as supplementary data at *Bioinformatics* online for a detailed derivation based on the Karush-Kuhn-Tucker conditions and the implicit function theorem. This property enables end-to-end supervised fine-tuning of PLMs. In practice, gradients are propagated from the UOT loss through the Sinkhorn updates into the PLM encoder, allowing the model to directly learn embedding geometries optimized for alignment accuracy.

#### 2.3.1 Fine-tuning dataset

For supervised fine-tuning, we constructed a custom dataset derived from the CATH v4.4.0 database (Orengo *et al*. 1997). Homologous pairs (positives) were sampled from the same CATH Homologous Superfamily, while non-homologous pairs (negatives) were sampled from different superfamilies. To prevent data leakage between training and evaluation, we applied a two-step redundancy-reduction protocol. First, we employed a group-aware splitting strategy, ensuring that all domains from the same superfamily were confined to a single data split. Second, we used MMseqs2 (Steinegger and Söding 2017) to filter out any sequences in the evaluation benchmarks (e.g. SABmark) that shared more than 30% sequence identity with our fine-tuning set. This process resulted in a final, non-redundant test set of 1,675 sequences. Ground-truth structural alignments for all pairs were generated using TM-align. (See Supplementary Section S5.1, available as supplementary data at *Bioinformatics* online for details).

#### 2.3.2 Training objective

The training objective uses a conditional loss function based on whether the input pair is positive (homologous) or negative (non-homologous).


$$L_{\text{total}}=\{\begin{array}{cc} L_{\text{positive}} & \text{if}\;\text{positive}\;\text{pair}\\ L_{\text{negative}} & \text{if}\;\text{negative}\;\text{pair} \end{array}$$

The individual loss components are defined as follows:

- For Positive Pairs: $L_{\text{positive}}=L_{\text{alignment}}+\lambda_{\text{pos}}\;L_{\text{sparsity}}$ with $\lambda_{\text{pos}}=0.1$.
- For Negative Pairs: $L_{\text{negative}}=\lambda_{\text{neg}}\;L_{\text{emptiness}}$ with $\lambda_{\text{neg}}=1.0$.

The primary alignment loss for positive pairs, $L_{\text{alignment}}$, is the Generalized KL divergence between the predicted plan Γ and the ground-truth plan *T*:


$$L_{\text{alignment}}=\text{KL}(T\|\Gamma ):=\sum_{i,j}\left(T_{ij}\;\text{log}\;\frac{T_{ij}}{\Gamma_{ij}}-T_{ij}+\Gamma_{ij}\right)$$

The regularization terms for positive ($L_{\text{sparsity}}$) and negative ($L_{\text{emptiness}}$) pairs are both based on the L1 norm of the unnormalized transport plan, $\sum_{i,j}|\Gamma_{ij}|$. For positive pairs, this encourages a sharp, sparse alignment path. For negative pairs, it forces the total mass of the transport plan towards zero, effectively preventing an alignment. Intuitively, this objective encourages confident alignments for homologous pairs while suppressing spurious correspondences for non-homologous ones.

#### 2.3.3 Hyperparameters

We performed supervised fine-tuning using the Low-Rank Adaptation (LoRA) method (Hu *et al*. 2021), which efficiently updates a small number of low-rank parameters while keeping the pretrained PLM weights frozen. Detailed hyperparameters for the LoRA configuration, optimizer, and training schedule are provided in Supplementary Section S5.2, available as supplementary data at *Bioinformatics* online.

### 2.4 Benchmark datasets for performance evaluation

We evaluated OTalign on three established remote-homology benchmarks—SABmark (Van Walle *et al*. 2005), MALIDUP (Cheng *et al*. 2007a), and MALISAM (Cheng *et al*. 2007b)—which together cover complementary aspects of protein sequence alignment under low similarity conditions (see Supplementary Section S3, available as supplementary data at *Bioinformatics* online for dataset statistics and descriptions).

SABmark contains over 29 000 protein pairs and is divided into two subsets: Superfamily and Twilight. The Superfamily subset includes sequences sharing probable common ancestry at the SCOP superfamily level, filtered at ≤50% sequence identity (average ≈ 18%), thus representing moderately remote homologs. The Twilight subset focuses on the extremely low similarity regime (average ≈ 11%), grouping single-domain proteins at the SCOP fold level with BLAST-based pairwise *E*-values ≥1.

MALIDUP consists of 241 homologous domain pairs generated through internal duplication within the same polypeptide chain, followed by substantial sequence divergence. These pairs provide clear cases of genuine homology despite low sequence similarity and include manually curated reference alignments with corresponding automatic alignments.

MALISAM comprises pairs of structurally similar but non-homologous motifs curated to evaluate false-positive control and to distinguish structural analogy from homology. As in MALIDUP, both manual reference alignments and automatic alignments are provided.

Together, these three benchmarks provide a balanced evaluation across remote homologous, duplicated, and analogous relationships, enabling comprehensive assessment of alignment performance under varying similarity levels. Implementation, parameters, and invocation of every method compared in Table 1 are listed in Supplementary Section S8, available as supplementary data at *Bioinformatics* online.

**Table 1:** Results on SABmark Superfamily and Twilight benchmarks.

Methods	**SABmark Superfamily**			**SABmark Twilight**		
	Precision	Recall	F1 score	Precision	Recall	F1 score
*Baselines*						
Needleman-Wunsch	0.300	0.386	0.334	0.100	0.150	0.118
HHalign	0.401	0.521	0.454	0.162	0.237	0.196
*PLM-based Frameworks*						
DeepBLAST (ProtT5-XL)	0.465	0.602	0.518	0.243	0.352	0.283
PLMAlign (ProtT5-XL)	0.371	0.694	0.469	0.183	0.448	0.253
OTalign (ProtT5-XL)	0.497	0.678	0.565	0.276	0.431	0.330
OTalign (ESM-1b)	0.367	0.500	0.417	0.157	0.249	0.189
OTalign (ESM-2 650M)	0.474	0.650	0.540	0.242	0.382	0.291
OTalign (Ankh-Large)	0.521	0.714	0.594	0.298	0.468	0.358

#### 2.4.1 Performance measurement

To evaluate alignment accuracy, we used three standard metrics—precision, recall, and F1-score—defined as:


$$\begin{array}{c} \text{Precision}=\frac{\text{TP}}{\text{TP}+\text{FP}},\;\text{Recall}=\frac{\text{TP}}{\text{TP}+\text{FN}}\\ F1\text{-score}=2\frac{\text{Precision}\cdot \text{Recall}}{\text{Precision}+\text{Recall}} \end{array}$$

Each residue pair in the predicted alignment is compared with the reference (ground-truth) alignment. TP (True Positive) are the number of residue pairs that are correctly aligned in both. FP (False Positive) are pairs aligned in the prediction but not in the reference. False Negatives (FN) are pairs aligned in the reference but missing in the prediction. Note that True Negatives (TN) are not considered due to the sparsity of aligned residue pairs. The F1-score provides a balanced measure of alignment accuracy by combining precision and recall.

## 3 Results and discussion

**Figure 1:**
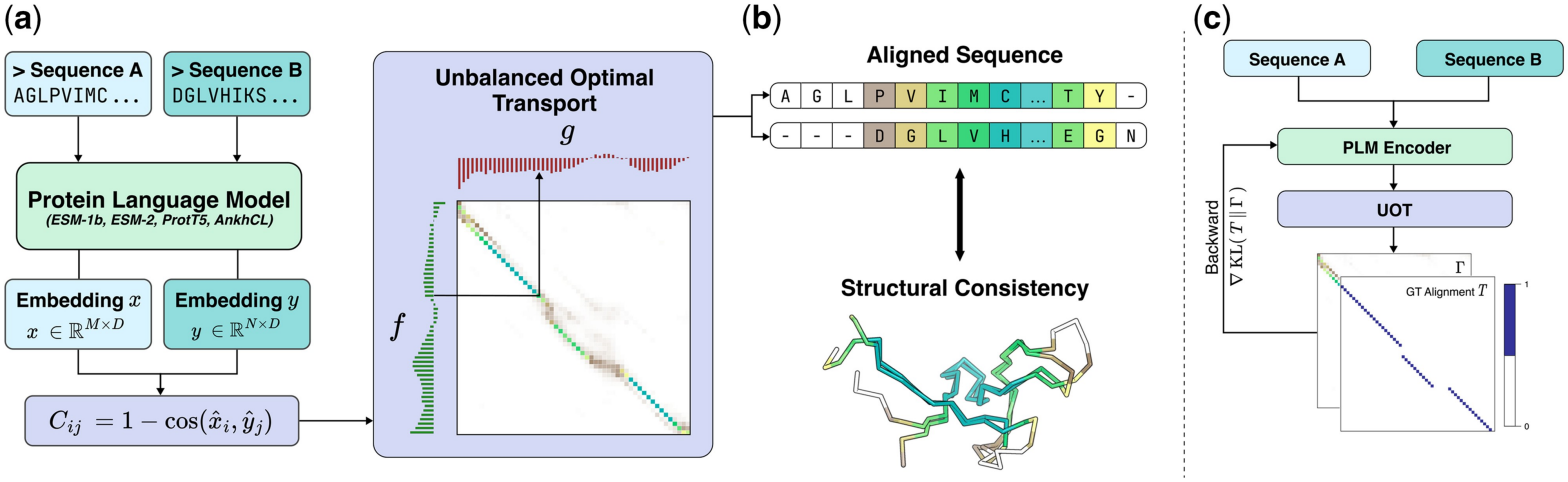
Overview of OTalign. (a) Two input sequences, *A* and *B*, are embedded into residue-level representations *x* and *y* by PLM. We then compute the pairwise cost matrix *C* and solve the entropy-regularized UOT problem to obtain the transport plan Γ. The panel shows a heatmap of Γ with the dual potentials *f* and *g* shown as bar plots (see Supplementary Section S1, available as supplementary data at *Bioinformatics* online). (b) Decoding the plan with a dynamic programming aligner yields a sequence alignment whose path is overlaid on the plan heatmap. The aligned structures are shown as backbone-only superpositions. (c) For supervised fine-tuning, a ground-truth plan *T* is derived from reference alignments. The KL divergence between *T* and Γ, KL(T‖Γ), defines the training loss, and its gradient is backpropagated through the UOT solver into the PLM parameters.

Figure 1 illustrates the overall OTalign pipeline. Given two protein sequences, a PLM encodes each into residue-level embeddings, from which a pairwise cost matrix is built and solved as an entropy-regularized UOT problem, producing a soft transport plan Γ and dual potentials *f* and *g* (Fig. 1a). A dynamic programming decoder then converts Γ into a discrete alignment path (Fig. 1b). For supervised fine-tuning, a ground-truth plan *T* from reference structural alignments is compared to Γ via KL divergence, and gradients are back-propagated through the UOT solver into the PLM (Fig. 1c). Below, we first benchmark OTalign on remote homology datasets and then probe how PLM embedding quality affects alignment accuracy. We also evaluate end-to-end fine-tuning gains, validate key design choices through ablation studies, and assess computational efficiency.

**Figure 2:**
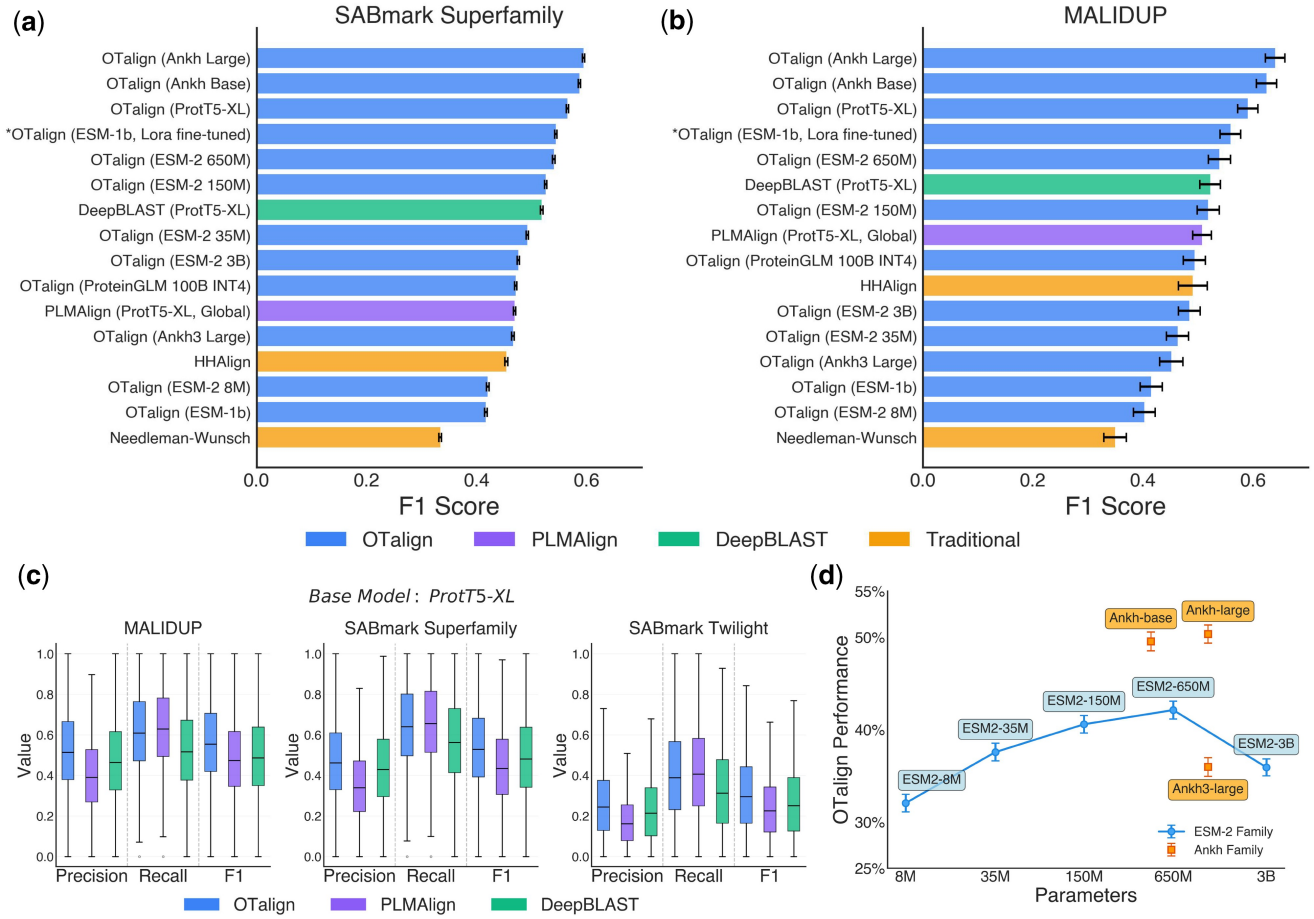
Comprehensive evaluation of OTalign performance. (a–b) F1 scores on SABmark Superfamily and MALIDUP datasets are shown in panels a and b, respectively. F1 scores obtained with OTalign variants are colored in blue, PLMAlign in purple, DeepBLAST in green, and traditional methods (Needleman-Wunsch and HHalign) in orange. Error bars denote 95% confidence intervals estimated from sample statistics across alignment pairs. The ESM-1b model fine-tuned with LoRA is annotated with an asterisk (*). Additional plots for MALISAM and SABmark Twilight are provided in Fig. S5, available as supplementary data at *Bioinformatics* online. (c) Comparison of OTalign, PLMAlign, and DeepBLAST using the shared ProtT5-XL embedding across MALIDUP, SABmark Superfamily, and Twilight datasets. OTalign shows a higher median F1, indicating more consistent alignment performance across diverse benchmarks. (d) Scaling analysis across the ESM-2 (blue) and Ankh (orange) model families under fixed OTalign settings. The *y*-axis represents the averaged alignment accuracy, computed as the mean of F1 scores from the MALIDUP and MALISAM datasets and Recall scores from the SABmark Superfamily and Twilight datasets. Error bars indicate the arithmetic mean of the standard errors from these datasets. Alignment accuracy does not necessarily increase with parameter count, and the observed differences cannot be fully explained by model size alone.

To evaluate how OTalign handles the challenging regime of remote homology, we benchmarked it against both traditional algorithms (Needleman–Wunsch, HHalign) and recent PLM-based methods (DeepBLAST, PLMAlign) using multiple PLM backends—ESM variants, Ankh variants, and ProtT5-XL. Table 1 summarizes the results on the SABmark Superfamily and Twilight benchmarks, and the bar plots in Fig. 2a and b visualize F1 scores across all tested methods. OTalign with Ankh-Large embeddings achieves the highest F1 scores on both SABmark subsets—0.594 on Superfamily and 0.358 on Twilight (Table 1)—outperforming all competing methods in precision, recall, and their harmonic mean. On the shared ProtT5-XL embedding, OTalign (F1 = 0.565 on Superfamily; 0.330 on Twilight) surpasses both PLMAlign (0.469; 0.253) and DeepBLAST (0.518; 0.283), demonstrating that the UOT-based framework extracts more alignment-relevant information from the same underlying representation. Beyond the SABmark datasets, OTalign also performs strongly on MALIDUP and MALISAM (Supplementary Tables S3 and S4, available as supplementary data at *Bioinformatics* online; bar plots for MALISAM and SABmark Twilight are shown in Fig. S5, available as supplementary data at *Bioinformatics* online). Specifically, OTalign with Ankh-Large achieves the best F1 score on MALIDUP (0.640), and on the challenging MALISAM dataset the ESM-1b LoRA fine-tuned variant attains the highest precision (0.187) and F1 (0.209), while PLMAlign with ProtT5-XL achieves the best recall (0.256).

### 3.1 Probing PLM representations via OTalign

Beyond alignment accuracy, OTalign also serves as a quantitative probe for assessing how well PLM embeddings capture biologically meaningful residue correspondences. A key question is whether alignment quality scales with PLM size, or whether other factors—such as pretraining objectives—play a dominant role. Because the OTalign decoder and its hyperparameters remain fixed across all experiments, any performance differences observed in Fig. 2a and b directly reflect differences in the underlying PLM embedding quality. Figure 2d presents a scaling analysis across the ESM-2 family (blue curve, 8M–3B parameters) and the Ankh family (orange curve, Ankh-Base to Ankh3-Large) under identical OTalign settings. The *y*-axis reports the averaged alignment accuracy (mean of MALIDUP/MALISAM F1 scores and SABmark Superfamily/Twilight recall scores). A striking finding is that alignment accuracy does not increase monotonically with model size. Within the ESM-2 series, performance peaks at 650M parameters and *decreases* at 3B (Fig. 2d, blue curve), suggesting that larger capacity alone does not guarantee better residue-level representations for alignment. Within the Ankh family, Ankh-Large outperforms the larger Ankh3-Large (Alsamkary *et al*. 2025) (Fig. 2d, orange curve). This discrepancy may partly reflect differences in pretraining objectives: Ankh uses a single denoising task, whereas Ankh3 employs a multi-task objective combining denoising and sequence completion, which may redistribute learned representations away from pairwise alignment-relevant features. Taken together, these results demonstrate that OTalign provides not only a high-performance alignment framework but also an interpretable diagnostic tool for evaluating the alignment suitability of PLM embeddings. A summary leaderboard of these comparative results is available at https://otalign.deepfold.org and in Supplementary Section S10, available as supplementary data at *Bioinformatics* online.

### 3.2 Effect of supervised fine-tuning

**Figure 3:**
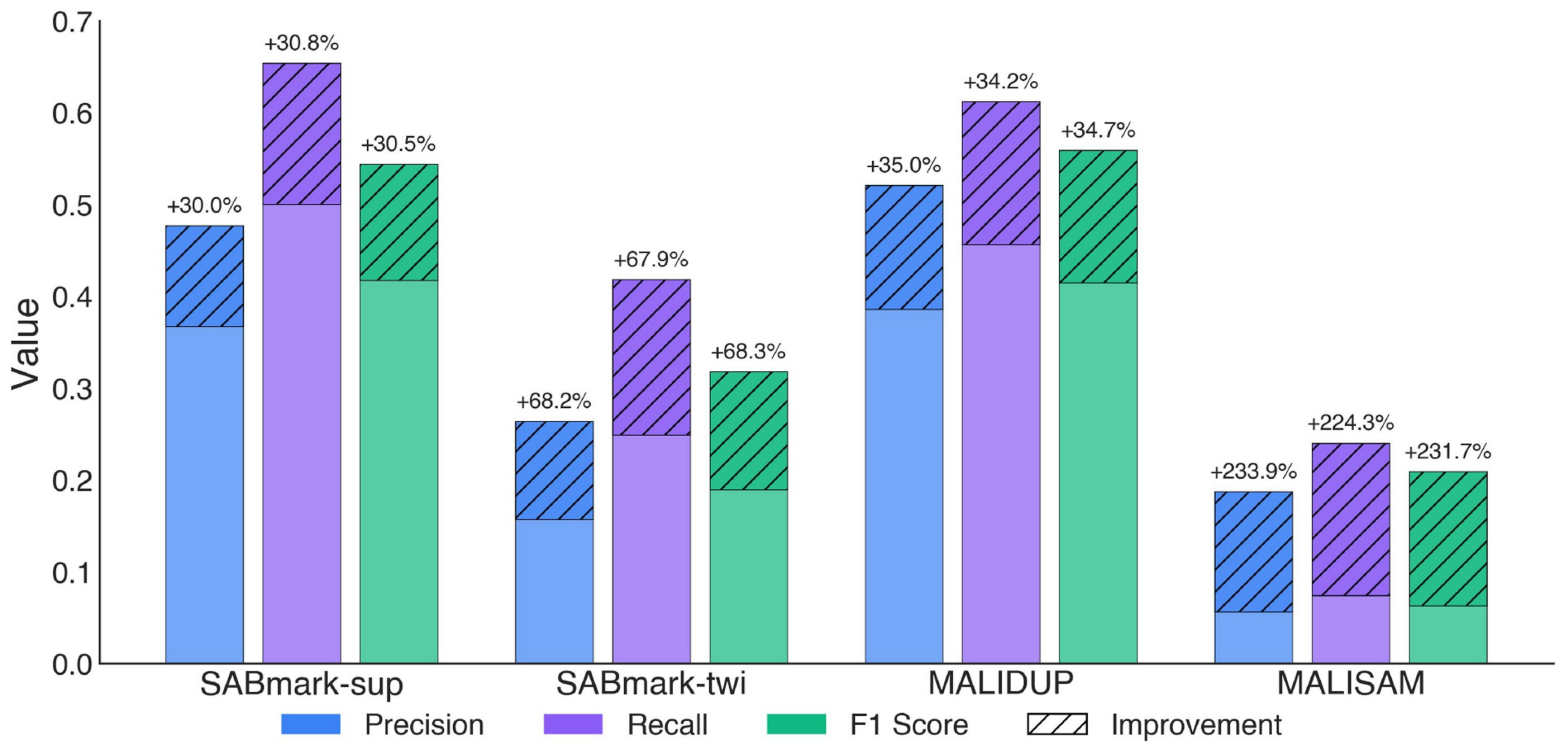
Performance gains from supervised fine-tuning. Solid bars show the baseline OTalign performance with the original ESM-1b encoder, and hatched segments indicate the absolute increase after LoRA-based fine-tuning on each benchmark (SABmark Superfamily, SABmark Twilight, MALIDUP, and MALISAM). Percentage values above the bars denote the corresponding relative improvements for precision, recall, and F1 score.

To test whether OTalign’s differentiable formulation can improve PLM representations end-to-end, we fine-tuned the ESM-1b encoder with LoRA (Hu *et al*. 2021) while keeping the OTalign decoder and all hyperparameters fixed (see Methods Sec. 2.3). Figure 3 summarizes the results: solid bars show baseline OTalign performance with the original ESM-1b encoder, and hatched segments indicate the absolute gains after fine-tuning. Fine-tuning simultaneously increased both precision and recall on every benchmark. This synergy yielded remarkable F1 improvements, from +30.5% on SABmark Superfamily (Fig. 3). Because the decoder was held fixed throughout, these improvements reflect genuine shifts in the embedding geometry—the PLM learns residue representations that better separate true correspondences from noise—rather than decoder overfitting.

### 3.3 Ablation studies validate design choices

To validate OTalign’s key design choices, we conducted ablation experiments focusing on the effects of UOT and the dual-potential formulation with SABmark Superfamily validation subset. The results, summarized in Table 2, demonstrate that both components are critical for achieving stable and biologically meaningful alignments. First, dynamic gap penalties derived from UOT dual potentials are critical for performance. Replacing our dynamic model with standard fixed gap penalties caused a drop in the F1 score by 4.7% (Exp 1). Furthermore, when constructing the dynamic penalties, using only the dual potentials (Exp 2b) achieved the full performance of the model, whereas using only the marginal mass signal (Exp 2a) offered no improvement over fixed penalties. This confirms that the dual potentials, which capture global alignment context, are the essential ingredient. Second, the UOT-derived match score matrix is central to alignment quality. Increasing the weight of the PMI-based match score led to a notable performance gain (Exp 3a), while decreasing it was detrimental (Exp 3b), highlighting its primary role in guiding the final alignment.

**Table 2:** Ablation study on the SABmark Superfamily validation set.

No.	Configuration	F1 score
–	OTalign (Full Model)	0.594 (0.0%)
*Gap Penalty Ablation*		
1	Fixed gap penalties	0.566 (−4.7%)
2a	Dynamic penalties (from mass only)	0.567 (−4.5%)
2b	Dynamic penalties (from duals only)	0.594 (0.0%)
*Match Score Ablation*		
3a	Increased match score weight (scale = 2.0)	0.600 (+1.0%)
3b	Decreased match score weight (scale = 0.5)	0.572 (−3.7%)

The study validates the two core components of the OTalign decoder: dynamic gap penalties derived from dual potentials and the PMI-based match score matrix.

### 3.4 Computational efficiency

We evaluated the computational cost of OTalign using an NVIDIA H100 NVL GPU (Fig. S3, available as supplementary data at *Bioinformatics* online). For batches of 50 sequence pairs, the end-to-end alignment time—including both Sinkhorn optimization (dashed lines in Fig. S3, available as supplementary data at *Bioinformatics* online) and dynamic programming decoding (difference between solid and dashed lines)—remained below 0.7 seconds across all tested sequence length products. The execution time scales as O(NM), consistent with the theoretical complexity of the cost matrix construction and iterative solver, yet GPU parallelization across batch elements keeps the absolute runtime practical even for long sequences. Detailed benchmarking protocols, hardware configurations, and additional runtime results are provided in Supplementary Section S7.2, available as supplementary data at *Bioinformatics* online.

### 3.5 Application: template realignment for structure prediction

To test whether OTalign’s alignment improvements translate into better *downstream* protein structure prediction, we applied it as a template realignment module within the ColabFold/AlphaFold 2 pipeline (Mirdita *et al*. 2022) on 17 CASP15 domains (detailed experimental setup in Supplementary Section S12, available as supplementary data at *Bioinformatics* online).

**Figure 4:**
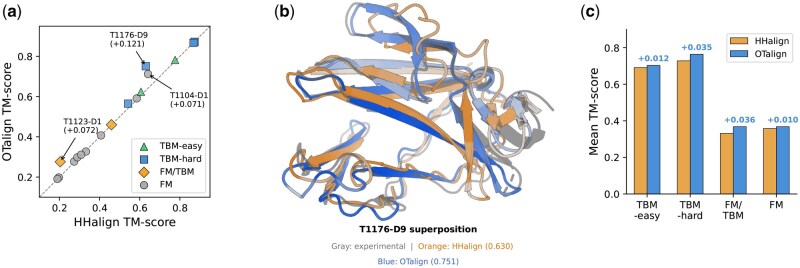
OTalign template realignment improves structure prediction on CASP15 domains. (a) Per-domain TM-score comparison between HHalign and OTalign template conditions across 17 CASP15 domains (pLDDT-best model selection). Points above the diagonal indicate domains where OTalign outperforms HHalign; 13 of 17 domains fall in this region (mean ΔTM = +0.019). The largest improvement is observed for T1176-D9 (+0.121), a TBM-hard domain where OTalign promotes the correct template (6XJ9_B) from rank 3 to rank 1. (b) Structural superposition of T1176-D9 predictions on the experimental structure (gray). Color saturation reflects per-residue Cα–Cα distance (darker = closer to reference). In the HHalign prediction (orange, TM = 0.630), the N-terminal β-strands (top of the structure) are displaced 3–5 Å from their native positions and fail to pack against the core β-sheet. In contrast, the OTalign prediction (blue, TM = 0.751) correctly positions these strands within 1–2 Å of the reference, recovering the native β-sandwich packing. (c) Mean TM-score by CASP difficulty category. OTalign yields consistent gains over HHalign for TBM-easy (+0.012) and TBM-hard (+0.035) categories, where template quality is the primary determinant of prediction accuracy.

Under practical model selection (pLDDT-best), OTalign realignment improved 13 of 17 domains, raising the mean TM-score from 0.481 to 0.501 (Δ=+0.019; Fig. 4a, Table S7, available as supplementary data at *Bioinformatics* online). The gains were strongest for TBM-hard domains (+0.035; Fig. 4c), where templates exist but sequence-based alignments are unreliable. The most striking case was T1176-D9, where OTalign promoted a closer structural homolog (6XJ9_B) to rank 1, yielding a TM-score gain of +0.121 (Fig. 4b). For FM domains lacking informative templates, neither method provided meaningful signal, confirming that OTalign enhances the *utilization* of existing structural information rather than creating signal where none exists (Supplementary Section S12). These results position OTalign as a practical plug-in for improving template-based structure prediction in the remote homology regime.

## 4 Conclusion

We presented OTalign, a differentiable alignment framework that applies unbalanced optimal transport to protein embeddings. It achieves state-of-the-art accuracy on remote homolog benchmarks while remaining interpretable and computationally efficient. On SABmark, OTalign achieves the best F1 score among tested methods, reflecting a more balanced precision–recall profile compared to PLM-based aligners. Scaling analyses revealed that alignment quality does not increase monotonically with model size. For the ESM-2 series, performance generally improved as parameter count increased, but the largest model, ESM-2 (3B), showed a decline in alignment accuracy. This suggests that despite its size, the 3B model may be less suited to the alignment task or not yet sufficiently optimized for capturing residue-level correspondences. A similar trend was observed in the Ankh family. While Ankh-Large outperformed Ankh-Base, the more complex Ankh3-Large—trained with a multi-task pre-training objective—exhibited degraded performance.

Leveraging OTalign’s fully differentiable formulation, we fine-tuned ESM-1b with LoRA, which improved both precision and recall across all benchmark sets, yielding consistent F1 gains. Because the decoder and hyperparameters were fixed, these improvements reflect shifts in embedding geometry rather than decoder overfitting. Note that MALISAM treats structural analogs as positives with curated reference alignments; thus, higher MALISAM F1 denotes better analog alignment, which is desirable for geometry-aware evaluation yet requires care when the goal is strict homology discrimination.

Furthermore, we demonstrated that OTalign’s alignment improvements propagate to a downstream biological application: when used as a template realignment module within ColabFold/AlphaFold 2, OTalign improved structure prediction quality on 13 of 17 CASP15 domains (mean ΔTM = +0.019, pLDDT-best selection) by reranking templates based on PLM-derived alignment scores, with gains of up to +0.121 TM-score on individual targets. This result validates that better sequence alignments translate into better structural models, particularly in the remote homology regime where OTalign’s advantages are most pronounced.

Looking ahead, we will integrate evolutionary cues (e.g. co-evolutionary information from MSAs) into the transport objective to prioritize conserved residues and better balance precision-recall for homolog detection with statistical significance. In parallel, we will leverage OTalign’s differentiable framework to develop models that learn sequence embeddings explicitly optimized for protein structure prediction (Lee *et al*. 2023, Kim *et al*. 2025) and structural alignment.

## Supplementary Material

btag476_Supplementary_Data

## Data Availability

The OTalign source code, configuration files, and benchmark pipeline are available at https://github.com/DeepFoldProtein/OTalign. All per-pair predictions, aggregated metrics, HMM profiles, and ECOD30 hard score matrices used to produce the figures and tables in this manuscript and supplement are deposited at Zenodo (https://zenodo.org/records/20407169).
