# Supplementary material for: OTalign: optimal transport alignment for remote protein homologs using protein language model embeddings
Source: Bioinformatics. 2026 Jun 30;42(7):btag476. doi: 10.1093/bioinformatics/btag476 (PMC13371769; doi:10.1093/bioinformatics/btag476)
Supplement: btag476_Supplementary_Data [file btag476_supplementary_data.pdf]

Supplementary Material for: OTalign: Optimal Transport Alignment for Remote Homologs with Protein Language Model Representations

Minsoo Kim, Hanjin Bae, Gyeongpil Jo, Kunwoo Kim, Jejoong Yoo,
and Keehyoung Joo

Contents

S1 Mathematical Details of the Differentiable Framework **2**
- S1.1 Differentiability via Entropy Regularization . . . 2
- S1.2 Dual Formulation and Optimality Conditions . . . 2
- S1.3 Implicit Differentiation of the Optimality Conditions . . . 3
- S1.4 Computational Complexity and Memory Profile . . . 3
- S1.5 Numerical Stability and Convergence . . . 4
- S1.6 Initialization and Stopping Criteria . . . 4

S2 Ablation on UOT Hyperparameters **4**

S3 Benchmark Dataset **4**

S4 Detailed Formulation of Position-Specific Gap Penalties **5**

S5 Fine-tuning Details **5**
- S5.1 Supervised Fine-tuning Dataset Construction . . . 5
- S5.2 Fine-tuning hyperparameters . . . 6

S6 Full Alignment Results **7**

S7 Homology Detection and Runtime Benchmarks **9**
- S7.1 Homology Detection and Discrimination Analysis . . . 9
- S7.2 Wall-Clock Runtime Benchmark . . . 10

S8 Baseline Methods and Invocation Details **12**

S9 Additional Benchmark Results: SABmark Twilight and MALISAM **15**

S10 OTalign Leaderboard Platform **16**

S11 CASP15 Per-Domain TM-Scores **18**

S12 CASP15 Template Realignment: Detailed Methods and Per-Category Analysis **19**
- S12.1 Experimental Setup . . . 19
- S12.2 Per-Category Analysis . . . 19
- S12.3 Biological Implications . . . 19

S1 Mathematical Details of the Differentiable Framework

A key advantage of our UOT formulation is its full differentiability with respect to the input embeddings, which enables end-to-end supervised fine-tuning. This differentiability is not achieved by unrolling the forward Sinkhorn iterations, which can be memory-intensive and numerically unstable. Instead, we compute the gradient via implicit differentiation of the optimality conditions of the UOT problem.

S1.1 Differentiability via Entropy Regularization

The entropy regularization term $\varepsilon \, \mathrm{KL}\big(\Gamma \,\|\, \mathbf{a}\mathbf{b}^\top\big)$ in the UOT objective is crucial. It renders the objective function strictly convex, which guarantees the existence of a unique, smooth optimal transport plan Γ^* [Feydy et al., 2019, Séjourné et al., 2023]. This smoothness ensures that the solution is a differentiable function of the input cost matrix C, which is a prerequisite for applying gradient-based optimization to the PLM embeddings that produce C. The Sinkhorn algorithm efficiently finds this unique solution by iteratively updating dual variables, which converge to a fixed point.

S1.2 Dual Formulation and Optimality Conditions

We consider the UOT problem in Eq. 1 (main text), with cost matrix C, uniform marginals $\mathbf{a}$ and $\mathbf{b}$, entropy parameter $\varepsilon > 0$, and marginal-relaxation parameter $\tau > 0$. Let $\lambda = \frac{\tau}{\tau+\varepsilon}$ and the Gibbs kernel $K_{ij} = \exp(C_{ij}/\varepsilon)$.

Dual potentials Introducing dual variables $f \in \mathbb{R}^M$ and $g \in \mathbb{R}^N$ for the relaxed marginal terms yields an unconstrained, strictly concave dual (f, g). Up to additive constants (independent of f, g), one convenient form is

$$\min_{f,g} \varepsilon \sum_{ij} a_i b_j \left[\exp\left(\frac{f_i + g_j - C_{ij}}{\varepsilon}\right) - 1\right] + \tau \sum_i a_i \left[\exp\left(-\frac{f_i}{\tau}\right) - 1\right] + \tau \sum_j b_j \left[\exp\left(-\frac{g_j}{\tau}\right) - 1\right],$$

whose stationarity conditions reproduce the Karush–Kuhn–Tucker (KKT) system [Boltyanski et al., 2013] used by Sinkhorn-type solvers. Intuitively, f and g act as position-wise "prices" for departing from the marginals, with their scales governed by τ and ε. The Sinkhorn algorithm therefore operates in the dual, not directly on the primal transport Γ.

KKT fixed points Setting the gradient of the dual to zero gives the coupled fixed-point equations

$$\exp(f_i) = \left(\frac{a_i}{\sum_{j=1}^N K_{ij} \exp(g_j)}\right)^\lambda \tag{S1}$$

$$\exp(g_j) = \left(\frac{b_j}{\sum_{i=1}^M K_{ij} \exp(f_i)}\right)^\lambda \tag{S2}$$

with $\lambda = \tau/(\tau + \varepsilon)$. Alternating these updates is precisely the UOT Sinkhorn iteration used in our method. Existence and uniqueness of the fixed point follow from strict convexity induced by the entropy term.

Recovering the plan At a stationary point (f^*, g^*), the optimal plan has the factorization

$$\Gamma_{ij} = a_i b_j \exp\left(\frac{f_i^* + g_j^* - C_{ij}}{\varepsilon}\right) \propto u_i K_{ij} v_j, \quad u_i := \exp(f_i^*), \quad v_j := \exp(g_j^*),$$

and satisfies row/column sums $\Gamma\mathbf{1}$, $\Gamma^\top\mathbf{1}$ that need not equal $(\mathbf{a}, \mathbf{b})$ when $\tau < \infty$ (unbalanced setting). In practice, the fixed-point scaling (u, v) is computed by iterating Eqs. S1 and S2.

S1.3 Implicit Differentiation of the Optimality Conditions

The KKT conditions (Eq. S1 and Eq. S2) can be abstracted as a single vector-valued function $H(f, g, C) = 0$, where finding the optimal duals is equivalent to finding the roots of H. The implicit function theorem states that if we can define such a function, we can compute the derivatives of the optimal variables with respect to the inputs without needing to differentiate through the iterative process that found them.

Adjoint system Differentiating $H(f, g, C) = 0$ gives

$$\begin{pmatrix} \frac{\partial H_f}{\partial f} & \frac{\partial H_f}{\partial g} \\ \frac{\partial H_g}{\partial f} & \frac{\partial H_g}{\partial g} \end{pmatrix} \begin{pmatrix} df \\ dg \end{pmatrix} + \frac{\partial H}{\partial C} : dC = 0.$$

Given a scalar loss $\mathcal{L}$ that depends on (f, g) and/or downstream quantities computed from them, the gradients with respect to (f, g) are obtained by solving the adjoint linear system

$$J(f, g)^\top \mu = t,$$

where $\mu = [\mu_f \mid \mu_g]$ are the adjoint variables and t is the vector assembled from $\partial\mathcal{L}/\partial f$ and $\partial\mathcal{L}/\partial g$ (as produced by the forward pass). We then obtain $\partial\mathcal{L}/\partial C$ by contracting μ with $\partial H/\partial C$. This procedure is what our `SinkhornUOT.backward` implements.

Jacobian structure The Jacobian inherits a simple block form,

$$J(f, g) = \begin{pmatrix} \frac{\partial H_f}{\partial f} & \frac{\partial H_f}{\partial g} \\ \frac{\partial H_g}{\partial f} & \frac{\partial H_g}{\partial g} \end{pmatrix} = \begin{pmatrix} I & \tau \mathrm{diag}(p)^{-1}\Gamma \\ \tau \mathrm{diag}(q)^{-1}\Gamma^\top & I \end{pmatrix}$$

where Γ is the optimal transport plan, and $p = \Gamma\mathbf{1}$ and $q = \Gamma^\top\mathbf{1}$ are its row and column marginals, respectively. This expression yields efficient Jacobian-vector products needed by the adjoint solver.

Computational remarks Although J is dense of size $(M + N) \times (M + N)$, we never form or invert it explicitly. Indeed, we solve the adjoint system with Schur-complement formulation and Conjugate Gradient, which only requires Jacobian-vector products implemented via matrix-vector operations with Γ, p, and q. This avoids the $O((M + N)^3)$ cost of direct factorization and keeps the backward pass aligned with the $O(MN)$ structure of the forward Sinkhorn.

S1.4 Computational Complexity and Memory Profile

Forward (UOT/Sinkhorn) Each iteration requires $O(MN)$ work to apply the Gibbs kernel $K_{ij} = \exp(-C_{ij}/\varepsilon)$ and update the dual potentials (f, g) via the fixed-point equations; the iteration count is typically modest under entropy regularization. Thus the forward pass is $O(T_{\mathrm{SK}}MN)$, where T_{SK} is the number of Sinkhorn steps.

Backward (Implicit Differentiation) We solve the adjoint linear system with a Schur-complement preconditioner and Conjugate Gradient (CG), avoiding explicit dense matrices; each CG iteration reduces to a sequence of matrix-vector products dominated by $O(MN)$. Overall, the backward pass scales as $O(T_{\mathrm{CG}}MN)$.

S1.5 Numerical Stability and Convergence

Safeguards We apply log-domain updates for f, g and the `log-sum-exp` trick to avoid overflow/underflow.

S1.6 Initialization and Stopping Criteria

Initialization We initialize $f = \log \mathbf{a}$, $g = \log \mathbf{b}$, which places updates near feasible marginals.

Stopping Criteria We terminate Sinkhorn when both the dual residual $\|f_{\text{new}} - f_{\text{old}}\|_1$ and $\|g_{\text{new}} - g_{\text{old}}\|_1$ fall below user tolerances; for CG we monitor the relative residual of the adjoint system.

S2 Ablation on UOT Hyperparameters

We also studied the UOT solver's hyperparameters.

Marginal Relaxation (τ) The model's performance was largely insensitive to the value of τ.

Entropy Regularization (ε) Performance peaked at $\varepsilon = 0.1$, likely because this level of entropy encourages sufficient spread in the transport plan to capture flexible alignments, without overly blurring correspondences. Empirically, it also achieves fast convergence in Sinkhorn iterations.

S3 Benchmark Dataset

Table S1: Summary of benchmark datasets.

Dataset	# Pairs	Mean Aligned Length (aa)	Mean Seq. Identity (%)
SABmark (Twilight)	10,667	65.15 ± 0.35	11.81 ± 0.06
SABmark (Superfamily)	19,092	91.68 ± 0.42	18.93 ± 0.08
MALIDUP	241	78.16 ± 2.54	18.00 ± 0.72
MALISAM	130	56.69 ± 1.00	8.54 ± 0.36

To assess alignment performance in the low-signal regime relevant to structure and function inference, we use three complementary resources: SABmark, MALIDUP, and MALISAM. Each targets a distinct failure mode of sequence-based methods and provides clear, reproducible ground truth.

SABmark Superfamily and Twilight SABmark samples the known fold space to evaluate aligners under low and very low similarity. It has two subsets. The twilight subset groups single-domain proteins at the SCOP fold level, with pairs selected by a BLAST-based criterion (large theoretical database) requiring pairwise E-values ≥ 1, focusing on cases with very weak sequence signal. The Superfamilies subset groups sequences at the SCOP superfamily level under a $\leq 50\%$ identity constraint to reflect probable common ancestry. Both subsets provide consensus reference alignments from structural-alignment agreement and FP-augmented variants that include non-homologous yet sequence-similar pairs. These enable joint evaluation of sensitivity to true homology and specificity against look-alikes.

MALIDUP and MALISAM MALIDUP (manual alignments of duplicated domains) contains 241 homologous domain pairs generated by internal duplication within the same polypeptide chain, followed by substantial divergence. Because common origin within one chain is well supported, these pairs represent genuine homology with attenuated sequence signal and test whether an aligner can recover evolutionary relationships despite large sequence differences. MALIDUP provides manual reference alignments that emphasize structural plausibility (structural cores, secondary-structure continuity, hydrogen-bonding networks, conserved sites) and companion automatic alignments for comparison. MALISAM (manual alignments for structurally analogous motifs) collects pairs of structurally similar yet non-homologous motifs to probe false-positive control and the distinction between homology and analogy. Pairs are curated under defined scenarios (hybrid–core, interface–core, artificial–natural) to minimize hidden common ancestry while maintaining strong structural resemblance. As in MALIDUP, MALISAM provides manual reference alignments and automatic alignments to assess specificity and robustness to structural convergence.

S4 Detailed Formulation of Position-Specific Gap Penalties

As mentioned in the main text, OTalign derives position-specific gap penalties from the UOT solution. While our ablation study indicated that the dual potentials are the most critical component, our full model combines signals from both the marginal mass distribution and the dual potentials for completeness.

Marginal Mass Component The marginal mass for each residue, $m_i^A = \sum_j \Gamma_{ij}$ for sequence A and $m_j^B = \sum_i \Gamma_{ij}$ for sequence B, reflects how much it participates in the alignment. A higher mass suggests a conserved residue that is less likely to be part of a gap. We normalize this mass and apply a clipping function:

$$\tilde{m}_k = \text{clip}\left(\frac{m_k}{\text{median}(m_{m>0})}, c_{\text{lower}}, c_{\text{upper}}\right) \tag{S3}$$

where k indexes residues in either sequence.

Dual Potential Component We incorporate the dual potentials $f \in \mathbb{R}^M$ and $g \in \mathbb{R}^N$, which represent the cost of inserting or deleting mass. We standardize these potentials (z') and transform them using a sigmoid function, $\sigma(-\kappa_z \cdot z')$, to map them to a penalty scaling factor.

Combined Formulation These two signals are combined into a final modulating factor F_k:

$$F_k = (\tilde{m}_k)^\gamma \cdot \sigma(-\kappa_z \cdot z'_k) \tag{S4}$$

where γ controls sensitivity to mass and κ_z controls sensitivity to the dual potential. This factor modulates base gap penalties ($\text{go}_{\text{base}}, \text{ge}_{\text{base}}$) to produce the final position-specific penalties:

$$\text{go}_k = \max(\text{ge}_{\text{base}}, \text{go}_{\text{base}} \cdot F_k) \tag{S5}$$

$$\text{ge}_k = \max(\eta \cdot \text{ge}_{\text{base}}, \text{ge}_{\text{base}} \cdot F_k) \tag{S6}$$

where η sets a minimum ratio for the extension penalty relative to the base penalty.

S5 Fine-tuning Details

S5.1 Supervised Fine-tuning Dataset Construction

For supervised fine-tuning, we constructed a custom dataset from the CATH v4.4.0 database [Orengo et al., 1997]. The detailed protocol is as follows:

- **Positive Samples**: Homologous pairs were sampled from within the same CATH Homologous Superfamily. These pairs are expected to have a meaningful structural and evolutionary relationship.
- **Negative Samples**: Non-homologous pairs were sampled from different CATH superfamilies. To ensure they represent true negatives, we filtered these pairs to have a TM-score below 0.2, indicating significant structural dissimilarity.
- **Redundancy Reduction and Leakage Prevention**: This is a critical step to ensure the model's robustness. We implemented a two-fold strategy:
  1. **Group-aware Splitting**: Domains from the same CATH superfamily were kept entirely within the same data split (train, validation, or test) to prevent the model from learning superfamily-specific features.
  2. **Sequence Identity Filtering**: We used MMseqs2 [Steinegger and Söding, 2017] to calculate sequence identity between our fine-tuning set and evaluation benchmarks (e.g., SABmark). We applied a strict 30% identity threshold; any sequence in the test set sharing more than 30% identity with the training data was removed. This ensured that our evaluation focuses on the "twilight zone" of remote homology.
- **Dataset Scale**: Following the filtering process, the final evaluation set consisted of 1,675 non-redundant sequences. This scale provides sufficient statistical power to validate the model's performance on diverse protein folds.
- **Ground Truth**: For all pairs, ground-truth structural alignments were generated using TM-align. These alignments served as the gold standard for calculating the alignment loss during training.

S5.2 Fine-tuning hyperparameters

See Table S2 for fine-tuning hyperparameters.

Table S2: Fine-tuning Hyperparameters for OTalign (ESM-1b).

**Parameter Group**	**Value**
Model Base	ESM-1b (650M)
Fine-tuning Method	LoRA
LoRA Configuration	
Rank (r)	8
Alpha (α)	16
Dropout	0.1
Target Modules	'query', 'key', 'value', 'dense'
Training	
Batch Size	64
Optimizer	AdamW
Learning Rate	1.0×10^{-4}
Epochs	2
UOT Parameters	
Entropy Regularization (ε)	0.1
Marginal Relaxation (τ)	1.0

S6 Full Alignment Results

Tables S3 and S4 present the detailed performance metrics for all key models across all major datasets.

Table S3: Performance Comparison: Representative Methods

Method	Dataset	Precision	Recall	F1-Score
Baselines				
NWalign	MALIDUP	0.331 ± 0.020	0.373 ± 0.021	0.349 ± 0.020
	MALISAM	0.060 ± 0.009	0.075 ± 0.011	0.066 ± 0.010
	SABmark-sup	0.300 ± 0.002	0.386 ± 0.003	0.334 ± 0.002
	SABmark-twi	0.100 ± 0.002	0.150 ± 0.002	0.118 ± 0.002
HHalign	MALIDUP	0.465 ± 0.025	0.518 ± 0.027	0.491 ± 0.026
	MALISAM	0.010 ± 0.005	0.011 ± 0.006	0.011 ± 0.005
	SABmark-sup	0.401 ± 0.003	0.521 ± 0.003	0.454 ± 0.003
	SABmark-twi	0.162 ± 0.003	0.237 ± 0.004	0.196 ± 0.003
DeepBLAST	MALIDUP	0.500 ± 0.018	0.553 ± 0.019	0.522 ± 0.019
	MALISAM	0.144 ± 0.014	0.160 ± 0.015	0.151 ± 0.014
	SABmark-sup	0.465 ± 0.002	0.602 ± 0.002	0.518 ± 0.002
	SABmark-twi	0.243 ± 0.002	0.352 ± 0.003	0.283 ± 0.003
PLMAlign (ProtT5-XL)	MALIDUP	0.423 ± 0.016	0.666 ± 0.018	0.507 ± 0.017
	MALISAM	0.126 ± 0.010	**0.256 ± 0.018**	0.168 ± 0.012
	SABmark-sup	0.371 ± 0.002	0.694 ± 0.002	0.469 ± 0.002
	SABmark-twi	0.183 ± 0.002	0.448 ± 0.003	0.253 ± 0.002
OTalign				
OTalign (Ankh-Large)	MALIDUP	**0.595 ± 0.018**	**0.703 ± 0.018**	**0.640 ± 0.018**
	MALISAM	0.171 ± 0.015	0.218 ± 0.018	0.191 ± 0.016
	SABmark-sup	**0.521 ± 0.002**	**0.714 ± 0.002**	**0.594 ± 0.002**
	SABmark-twi	**0.298 ± 0.002**	**0.468 ± 0.003**	**0.358 ± 0.003**
OTalign (ESM-1b)	MALIDUP	0.386 ± 0.020	0.456 ± 0.021	0.415 ± 0.020
	MALISAM	0.056 ± 0.009	0.074 ± 0.012	0.063 ± 0.010
	SABmark-sup	0.367 ± 0.002	0.500 ± 0.002	0.417 ± 0.002
	SABmark-twi	0.157 ± 0.002	0.249 ± 0.003	0.189 ± 0.002
OTalign (ProtT5-XL)	MALIDUP	0.550 ± 0.018	0.646 ± 0.019	0.590 ± 0.018
	MALISAM	0.180 ± 0.015	0.230 ± 0.019	0.201 ± 0.017
	SABmark-sup	0.497 ± 0.002	0.678 ± 0.002	0.565 ± 0.002
	SABmark-twi	0.276 ± 0.002	0.431 ± 0.003	0.330 ± 0.003
OTalign (ESM-1b, LoRA full)	MALIDUP	0.521 ± 0.019	0.612 ± 0.019	0.559 ± 0.019
	MALISAM	**0.187 ± 0.015**	0.240 ± 0.019	**0.209 ± 0.016**
	SABmark-sup	0.477 ± 0.002	0.654 ± 0.002	0.544 ± 0.002
	SABmark-twi	0.264 ± 0.002	0.418 ± 0.003	0.318 ± 0.003

Table S4: Performance Analysis: Additional OTalign Variants

Method	Dataset	Precision	Recall	F1-Score
OTalign (ESM-2 35M)	MALIDUP	0.431 ± 0.020	0.507 ± 0.021	0.463 ± 0.020
	MALISAM	0.095 ± 0.011	0.126 ± 0.015	0.108 ± 0.013
	SABmark-sup	0.432 ± 0.002	0.591 ± 0.002	0.492 ± 0.002
	SABmark-twi	0.216 ± 0.002	0.341 ± 0.003	0.259 ± 0.003
OTalign (ESM-2 150M)	MALIDUP	0.483 ± 0.020	0.569 ± 0.021	0.519 ± 0.020
	MALISAM	0.095 ± 0.011	0.124 ± 0.014	0.107 ± 0.012
	SABmark-sup	0.462 ± 0.002	0.631 ± 0.002	0.525 ± 0.002
	SABmark-twi	0.233 ± 0.002	0.367 ± 0.003	0.280 ± 0.003
OTalign (ESM-2 650M)	MALIDUP	0.502 ± 0.020	0.591 ± 0.020	0.539 ± 0.020
	MALISAM	0.101 ± 0.012	0.131 ± 0.015	0.113 ± 0.013
	SABmark-sup	0.474 ± 0.002	0.650 ± 0.002	0.540 ± 0.002
	SABmark-twi	0.242 ± 0.002	0.382 ± 0.003	0.291 ± 0.003
OTalign (ESM-2 3B)	MALIDUP	0.451 ± 0.020	0.532 ± 0.020	0.484 ± 0.020
	MALISAM	0.068 ± 0.010	0.089 ± 0.013	0.076 ± 0.011
	SABmark-sup	0.418 ± 0.002	0.572 ± 0.002	0.476 ± 0.002
	SABmark-twi	0.191 ± 0.002	0.304 ± 0.003	0.231 ± 0.002
OTalign (ESM-2 8M)	MALIDUP	0.377 ± 0.019	0.438 ± 0.020	0.402 ± 0.020
	MALISAM	0.099 ± 0.012	0.129 ± 0.015	0.111 ± 0.013
	SABmark-sup	0.370 ± 0.002	0.503 ± 0.002	0.420 ± 0.002
	SABmark-twi	0.169 ± 0.002	0.266 ± 0.003	0.203 ± 0.002
OTalign (Ankh3-Large)	MALIDUP	0.425 ± 0.021	0.487 ± 0.022	0.451 ± 0.021
	MALISAM	0.114 ± 0.012	0.144 ± 0.015	0.126 ± 0.014
	SABmark-sup	0.411 ± 0.002	0.557 ± 0.002	0.466 ± 0.002
	SABmark-twi	0.193 ± 0.002	0.303 ± 0.003	0.231 ± 0.002
OTalign (Ankh-Base)	MALIDUP	0.582 ± 0.019	0.684 ± 0.019	0.625 ± 0.019
	MALISAM	0.175 ± 0.015	0.220 ± 0.019	0.194 ± 0.017
	SABmark-sup	0.515 ± 0.002	0.706 ± 0.002	0.587 ± 0.002
	SABmark-twi	0.291 ± 0.002	0.458 ± 0.003	0.349 ± 0.003
OTalign (ProteinGLM 100B INT4)	MALIDUP	0.460 ± 0.020	0.540 ± 0.020	0.494 ± 0.020
	MALISAM	0.067 ± 0.011	0.087 ± 0.013	0.075 ± 0.012
	SABmark-sup	0.415 ± 0.002	0.564 ± 0.002	0.471 ± 0.002
	SABmark-twi	0.194 ± 0.002	0.304 ± 0.003	0.233 ± 0.002

S7 Homology Detection and Runtime Benchmarks

S7.1 Homology Detection and Discrimination Analysis

We evaluate remote homology detection on the ECOD30 hard benchmark [Kaminski et al., 2023] (3,000 positive and 1,115,675 negative pairs at $\leq 30\%$ sequence identity) and compare OTalign against pLM-BLAST, EBA [Pantolini et al., 2024], and HHalign [Söding, 2004] (Section S8 for method-level configuration). Note that OTalign is primarily a residue-level alignment tool, whereas the three baselines are optimized specifically for homology detection. Figure S1 shows that OTalign is competitive with the dedicated PLM-based detection methods and that neither HHalign mode exceeds the embedding-based methods. ECOD H-group membership was constructed in part with HHsearch [Kaminski et al., 2023], so HHsearch-derived methods may benefit from a degree of circularity on this particular benchmark.

Figure S2 reconciles the MALISAM alignment-quality result with the homology-detection result above: using one identical OTalign configuration across all three groups, structurally analogous pairs are scored systematically below true homologs while still well above unrelated pairs. OTalign therefore *aligns* analogous pairs plausibly when asked to do so but *scores* them as non-homologous, consistent with the homology-detection comparison.

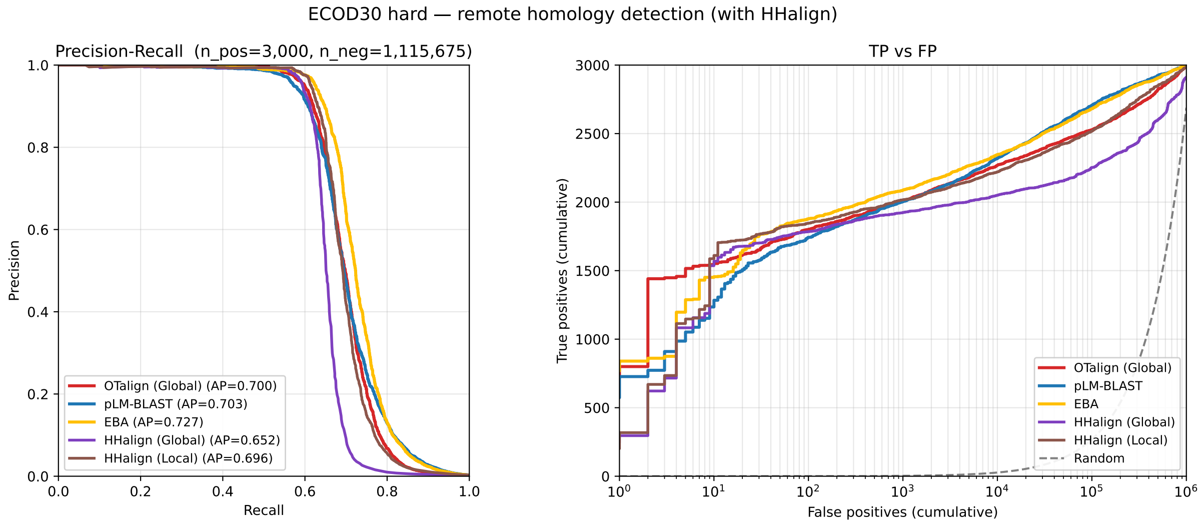

Figure S1: **Remote homology detection on the ECOD30 hard dataset, including HHsearch/HHalign.** Precision–recall (left) and cumulative TP-vs-FP curves (right; semi-log, x-axis to 10^6 false positives) for OTalign (red), pLM-BLAST (blue), EBA (gold), HHalign `-global 1` (purple), and HHalign default local (brown). Grey: random baseline. ROC-AUC / PR-AUC: pLM-BLAST 0.963 / 0.703; EBA 0.960 / 0.727; HHalign-local 0.937 / 0.696; OTalign 0.932 / 0.700; HHalign-global 0.874 / 0.652. Method-level configuration is given in Section S8.

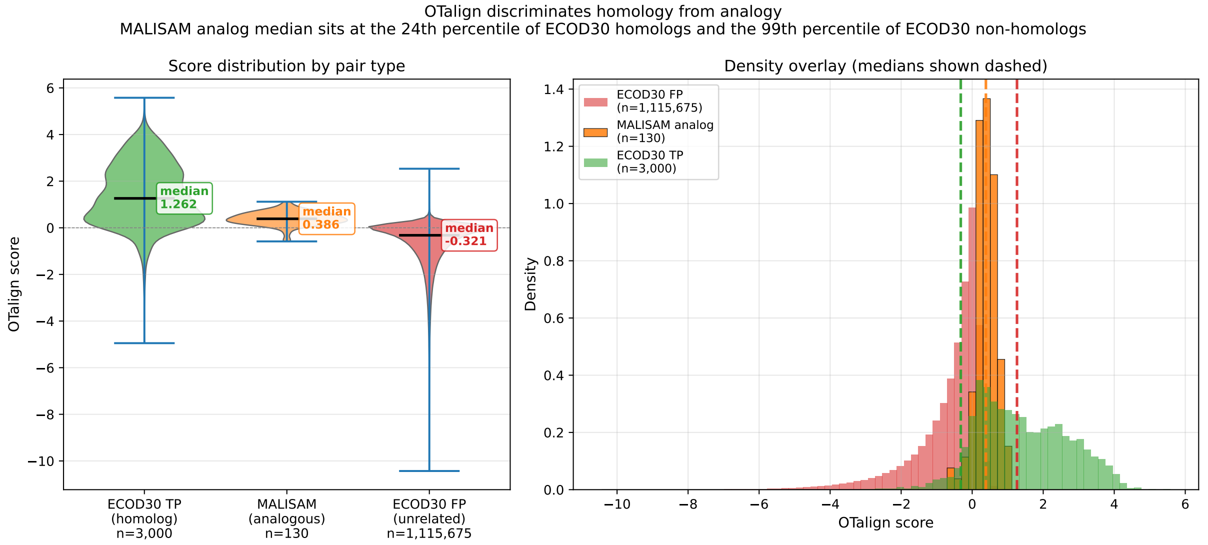

Figure S2: **OTalign systematically scores structurally analogous pairs below true homologs.** Length-normalized OTalign scores (Ankh-Large, global DP, identical pipeline across datasets) for ECOD30 true homologs (same H-group; $n = 3{,}000$, blue), MALISAM structurally analogous but evolutionarily unrelated pairs ($n = 130$, orange), and ECOD30 unrelated pairs (different X-group; $n = 1{,}115{,}675$, grey). Violin (left) and density overlay (right); medians annotated. The median MALISAM analog score (0.39) lies at the 24th percentile of true ECOD30 homologs and the 99th percentile of unrelated ECOD30 pairs.

S7.2 Wall-Clock Runtime Benchmark

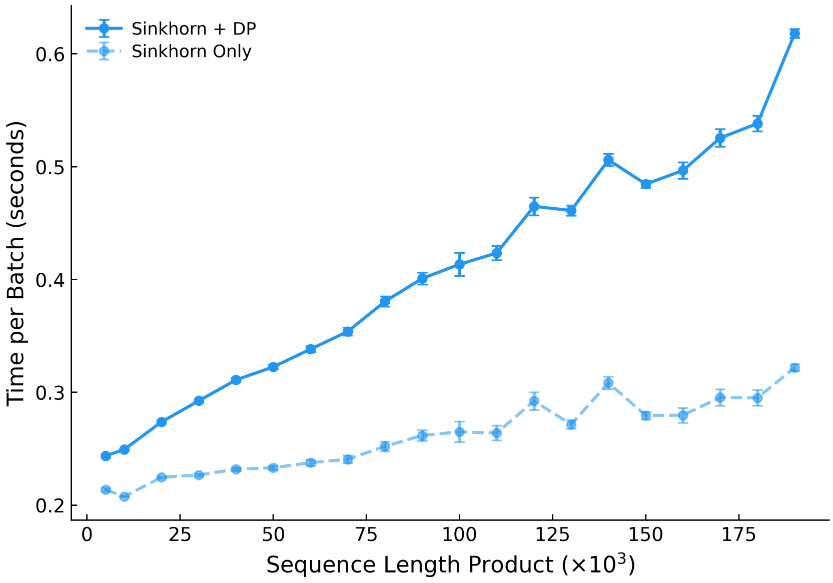

Figure S3: **Wall-Clock Runtime Benchmark Results.** Wall-clock runtime benchmark using PLM embeddings at batch size 50. Solid lines show the full pipeline (Sinkhorn followed by DP); dashed lines show Sinkhorn only. Error bars indicate the standard error computed from 10 independent batch runs

Data Preparation We benchmarked the wall-clock runtime of OTalign using residue-level embeddings for the SABmark dataset generated via ESM-2 650M. Protein pairs were grouped into 20 bins based on the product of the sequence lengths ($M \times N$) in each pair.

Measurement Protocol All benchmarks were conducted on an NVIDIA H100 NVL GPU (94 GB VRAM) using FP32 precision with PyTorch. Timing was recorded for two distinct phases: (i) the end-to-end pipeline, encompassing both Sinkhorn optimization and dynamic programming alignment, and (ii) the Sinkhorn computation alone to isolate the optimization cost. For each bin, we measured the computation time for batches of 50 pairs and averaged the results over 10 independent runs to calculate the mean and standard error.

Results As shown in Figure S3, the computation time increases with sequence length, scaling as $O(NM)$ due to the pairwise cost matrix construction and Sinkhorn iterations. Despite this quadratic scaling, GPU parallelization across batch elements keeps the absolute runtime practical, processing 50 pairs in under 1 second for all tested sequence lengths. Additionally, the dynamic programming alignment step also exhibits $O(NM)$ complexity and shows a similar increase in computation time with sequence length.

S8 Baseline Methods and Invocation Details

This section lists the implementation, version, and key parameters used for every method compared against OTalign. The reproducible end-to-end configuration is the file `configs/benchmark_config.yaml` (see the Data availability statement in the main text for the repository and data deposit). We summarize only what is needed to interpret the reported numbers.

Table S5: Compared methods. Per-method invocation details are described in the paragraphs that follow; full hyperparameters live in `configs/benchmark_config.yaml` of the public repository.

Method	Encoder / profiles	Mode
NWalign	BLOSUM (sequence only)	global
HHalign	HHblits HHM profile	default `-global 1` (local also evaluated)
DEDAL	DEDAL v3 (TF-Hub)	Smith–Waterman
DeepBLAST	ProtT5 (built-in)	Needleman–Wunsch
PLMAlign	ProtT5-XL/UniRef50	global
pLM-BLAST	ProtT5-XL/UniRef50	global
EBA	ProtT5	DTW + similarity-matrix reg. ℓ=1.0
OTalign	PLM (varied)	UOT + global DP (ε=0.1, τ=1.0)

NWalign (Needleman–Wunsch) The Zhang-group NWalign binary (https://aideepmed.com/NW-align/) was invoked on FASTA-formatted inputs (`-infmt1 4 -infmt2 4 -glocal 0`). NWalign serves as a pure-sequence reference using BLOSUM-style scoring.

HHalign (HMM–HMM profile alignment) Single-sequence FASTA inputs were converted to profile HMMs with HHblits [Remmert et al., 2012] run against the UniRef30 (release 2023_02) clustered sequence database (`hhblits -e 1e-3 -maxseq 65535`); the resulting A3M alignments were converted to HHM profiles with `hhmake -M first`. All HH-suite executables were invoked from the HH-suite 3.3.0 Singularity image packaged with our repository. For the alignment-quality benchmarks reported in main-text Table 1 (MALIDUP, MALISAM, SABmark), HHalign [Söding, 2004] was invoked as `hhalign -i q.hhm -t t.hhm -o pair.hhr -global 1`, with predicted residue–residue pairs extracted from the HHR detailed alignment block. The default local mode was additionally evaluated on the same benchmarks; both modes, together with conditional F1 (restricted to non-empty alignments), are reported in Table S6 and Fig. S4. For homology detection on ECOD30 hard (Fig. S1) the first hit's posterior probability (`Probab`) was used as the score, and both modes are reported there as well. The general properties of HHalign's local and global HMM–HMM alignment modes are described in the original HHsearch reference [Söding, 2004] and the HH-suite3 paper [Steinegger et al., 2019].

DEDAL We used DEDAL v3 from TensorFlow Hub (https://tfhub.dev/google/dedal/3) [Llinares-López et al., 2022] with `max_length=512` and GPU inference. DEDAL produces residue-pair Smith–Waterman scores from a learned encoder.

DeepBLAST We used the official DeepBLAST checkpoint `deepblast-v3.ckpt` [Hamamsy et al., 2023], configured as the Needleman–Wunsch decoder over its built-in ProtT5 encoder.

PLMAlign and pLM-BLAST Both methods use a ProtT5-XL/UniRef50 embedding cache that we build once with our `build_cache` script and share across the two evaluators. PLMAlign [Liu et al., 2024] and pLM-BLAST [Kaminski et al., 2023] were both run in their global alignment mode for consistency with OTalign's global regime.

EBA We used the official EBA implementation [Pantolini et al., 2024] with the ProtT5 encoder and similarity-matrix regularization ℓ=1.0.

OTalign (this work) All OTalign rows use the same decoder and hyperparameters across PLM backbones (Ankh-Large, Ankh-Base, Ankh3-Large, ProtT5-XL/UniRef50, ESM-1b, ESM-2 at 8M/35M/150M/650M/3B): entropy regularisation $\varepsilon = 0.1$, marginal-relaxation parameter $\tau = 1.0$, 1,000 Sinkhorn iterations, and a global Needleman–Wunsch DP decoder over the PMI-scored transport plan with dual-potential-derived gap penalties (base gap-open $= 8.0$, base gap-extend $= 1.0$, extend-floor ratio $\eta = 0.25$; dual sensitivity $k_f = k_g = 0.75$; mass-clipping range $[0.25, 4.0]$; PMI score scale $\alpha = 1.0$). The fine-tuned OTalign row in Table S3 uses the same decoder over the LoRA-tuned ESM-1b encoder (see Section S5.1). The ECOD30-hard ranking score is `mean_cosine` of the transport plan with $\lambda_1 = \lambda_2 = 1.0$.

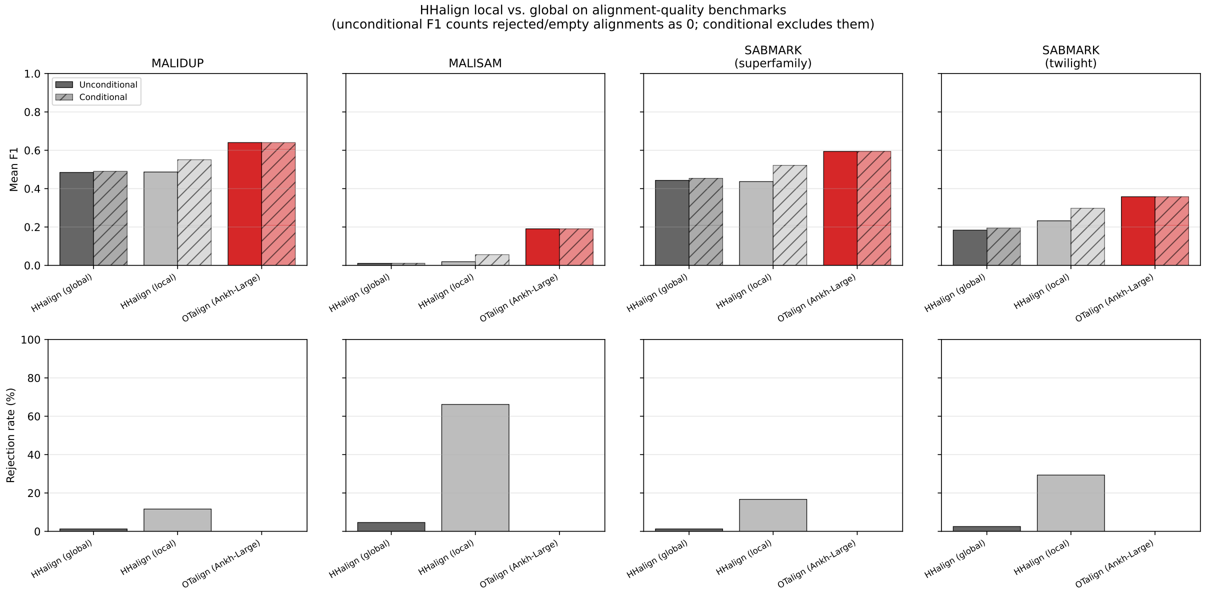

Figure S4: **HHalign mode and rejection behavior on alignment-quality benchmarks.** Top row: mean F1 per method on each alignment-quality benchmark, computed both unconditionally (solid bars; pairs with no predicted alignment contribute F1$=0$) and conditionally (hatched bars; only pairs with non-empty predicted alignments are scored). Bottom row: per-method rejection rate, defined as the fraction of pairs with `pred_size`$= 0$. HHalign was run in its default local HMM–HMM alignment mode (`-loc`, with MAC realignment enabled) and a forced global mode (`-global 1`). OTalign (Ankh-Large) is shown for reference.

Table S6: HHalign local vs. global mode on alignment-quality benchmarks. Conditional F1 is restricted to pairs where HHalign produced a non-empty alignment; OTalign (Ankh-Large) has no rejections.

Dataset	Method	n	Empty (%)	Uncond. F1	Cond. F1
MALIDUP	HHalign (global)	241	1.2	0.484	0.491
	HHalign (local)	241	11.6	0.487	0.551
	OTalign (Ankh-L)	241	0.0	**0.640**	**0.640**
MALISAM	HHalign (global)	130	4.6	0.010	0.011
	HHalign (local)	130	66.2	0.019	0.056
	OTalign (Ankh-L)	130	0.0	**0.191**	**0.191**
SABmark-sup	HHalign (global)	19,092	1.3	0.443	0.454
	HHalign (local)	19,092	16.7	0.437	0.521
	OTalign (Ankh-L)	19,092	0.0	**0.594**	**0.594**
SABmark-twi	HHalign (global)	10,667	2.5	0.184	0.195
	HHalign (local)	10,667	29.3	0.232	0.298
	OTalign (Ankh-L)	10,667	0.0	**0.358**	**0.358**

S9 Additional Benchmark Results: SABmark Twilight and MALISAM

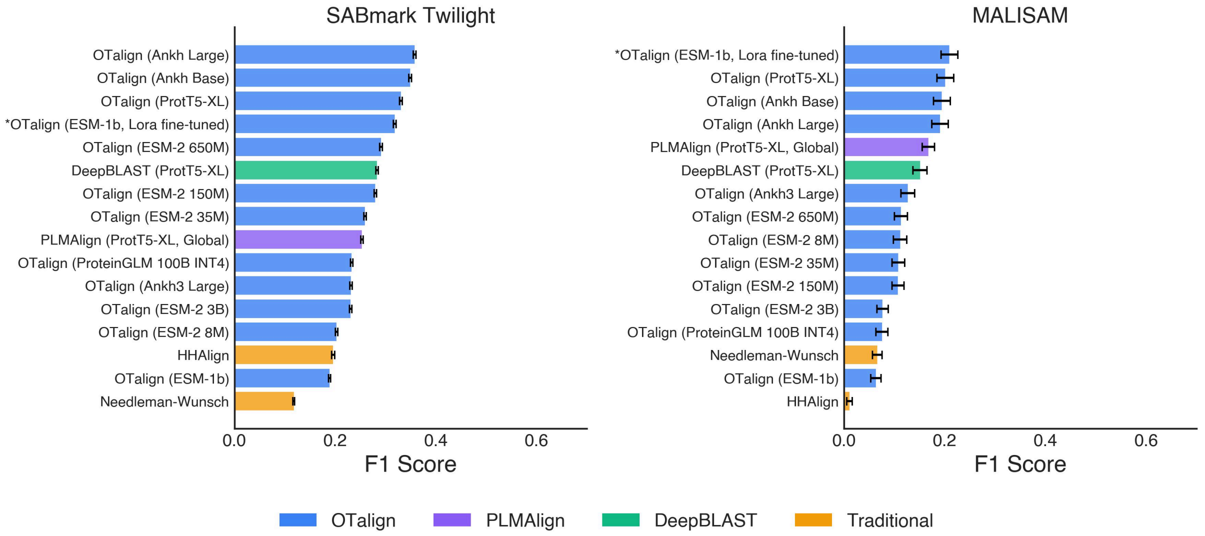

Figure S5: **Supplementary Benchmark Performance.** F1 scores on the SABmark Twilight and MALISAM datasets are shown in panels a and b, respectively. OTalign variants are shown in blue, PLMAlign in purple, DeepBLAST in green, and traditional methods (Needleman–Wunsch and HHalign) in orange. Error bars indicate 95% confidence intervals estimated from sample variation across alignment pairs. Models fine-tuned with LoRA are annotated with an asterisk (*). Detailed numerical results corresponding to these plots are provided in Tables S3 and S4.

S10 OTalign Leaderboard Platform

To promote transparent evaluation and community contributions, we provide a public platform[1] which hosts benchmark results for a broad range of PLMs and alignment methods. This resource enables systematic comparison, continuous updates, and collaborative analysis of PLM representations for alignment.

The main interface of the platform is shown in Fig. S6. The platform consists of four primary components: (1) **Leaderboard**: compares OTalign and various PLM-based methods across multiple benchmark datasets. (2) **Analysis**: visualizes scaling behavior between parameter count and performance, and provides model-specific performance profiles. (3) **Datasets**: summarizes benchmark datasets such as MALIDUP, MALISAM, and SABmark. (4) **Submission**: allows researchers to upload their methods and submit standardized JSON files for reproducible benchmarking.

[1] https://otalign.deepfold.org/

OTalign
Protein Alignment Benchmark

17 models Updated 10/1/2025

Leaderboard | Analysis | Datasets | Submit

Protein Alignment Leaderboard

Comparing alignment methods across challenging benchmark datasets

Sorted by F1 Score

Filter by type: All Types

Showing 17 of 17 entries

RANK	MODEL	TYPE	PARAMETERS	AVG	MALIDUP F1	MALISAM F1	SABMARK (SUP) RECALL	SABMARK (TWI) RECALL
1	OTalign (Ankh Large)	OTalign	1.15B	0.5034	0.6403	0.1906	0.7145	0.4683
2	OTalign (AnkhCL)	OTalign	1.15B	0.5027	0.6396	0.1911	0.7139	0.4660
3	OTalign (Ankh Base)	OTalign	450M	0.4955	0.6246	0.1941	0.7059	0.4576
4	OTalign (ProtT5-XL)	OTalign	3B	0.4753	0.5904	0.2011	0.6783	0.4313
5	OTalign (ESM-1b, LoRA full)	OTalign	650M	0.4602	0.5591	0.2094	0.6544	0.4180
6	PLMAlign (ProtT5-XL, Global)	PLM-based	3B	0.4541	0.5075	0.1675	0.6935	0.4481
7	OTalign (ESM-2 650M)	OTalign	650M	0.4212	0.5391	0.1133	0.6499	0.3824
8	DeepBLAST (ProtT5-XL)	PLM-based	3B	0.4067	0.5222	0.1508	0.6023	0.3516
9	OTalign (ESM-2 150M)	OTalign	150M	0.4058	0.5187	0.1070	0.6310	0.3666
10	OTalign (ESM-2 35M)	OTalign	35M	0.3757	0.4629	0.1078	0.5914	0.3408
11	OTalign (Ankh3 Large)	OTalign	1.15B	0.3596	0.4515	0.1264	0.5574	0.3031
12	OTalign (ProteinGLM 100B INT4)	OTalign	100B INT4	0.3593	0.4935	0.0754	0.5637	0.3045
13	OTalign (ESM-2 3B)	OTalign	3B	0.3592	0.4844	0.0764	0.5718	0.3041
14	OTalign (ESM-2 8M)	OTalign	8M	0.3205	0.4024	0.1114	0.5025	0.2657
15	HHAlign	Traditional	N/A	0.3148	0.4906	0.0110	0.5210	0.2366
16	OTalign (ESM-1b)	OTalign	650M	0.3069	0.4149	0.0633	0.5000	0.2492
17	Needleman-Wunsch	Traditional	N/A	0.2378	0.3492	0.0662	0.3861	0.1496

Figure S6: **OTalign leaderboard interface.** The leaderboard compares different alignment methods across MALIDUP, MALISAM, and SABmark datasets. It highlights OTalign variants alongside PLM-based and traditional algorithms, enabling direct evaluation of both sensitivity and specificity.

S11 CASP15 Per-Domain TM-Scores

Table S7: Per-domain TM-scores for all 17 CASP15 domains under three conditions. For each domain, we report TM-scores under both oracle selection (highest TM-score among 5 models) and practical selection (model with highest pLDDT). All predictions use single-sequence input with HHalign-derived templates; OTalign condition additionally realigns template sequences. Δ = OTalign − HHalign.

		Oracle (best TM)			pLDDT-best		
Domain	Class	HHalign	OTalign	Δ	HHalign	OTalign	Δ
T1137s4-D1	TBM-easy	0.776	0.782	+0.006	0.776	0.782	+0.006
T1145-D1	TBM-easy	0.606	0.627	+0.022	0.606	0.624	+0.018
T1119-D1	TBM-hard	0.873	0.872	–0.001	0.873	0.872	–0.001
T1121-D2	TBM-hard	0.542	0.565	+0.023	0.542	0.565	+0.023
T1152-D1	TBM-hard	0.868	0.867	–0.001	0.868	0.867	–0.001
T1176-D9	TBM-hard	0.630	0.751	**+0.120**	0.630	0.751	**+0.120**
T1106s1-D1	FM/TBM	0.576	0.543	–0.033	0.459	0.460	+0.000
T1123-D1	FM/TBM	0.326	0.316	–0.010	0.204	0.276	+0.073
T1104-D1	FM	0.716	0.712	–0.003	0.641	0.712	+0.071
T1120-D1	FM	0.585	0.591	+0.005	0.585	0.591	+0.005
T1121-D1	FM	0.296	0.315	+0.019	0.273	0.277	+0.004
T1122-D1	FM	0.402	0.392	–0.010	0.289	0.298	+0.009
T1137s3-D2	FM	0.221	0.217	–0.004	0.197	0.198	+0.001
T1137s4-D3	FM	0.401	0.394	–0.006	0.332	0.327	–0.005
T1137s6-D2	FM	0.224	0.221	–0.003	0.190	0.191	+0.001
T1159-D1	FM	0.415	0.408	–0.007	0.308	0.311	+0.004
T1187-D1	FM	0.418	0.484	+0.066	0.407	0.407	–0.001
**Mean**		0.522	0.533	+0.011	0.481	0.501	**+0.019**

S12 CASP15 Template Realignment: Detailed Methods and Per-Category Analysis

S12.1 Experimental Setup

We applied OTalign as a template realignment module within the ColabFold/AlphaFold2 pipeline [Mirdita et al., 2022] on 17 CASP15 domains spanning free modeling (FM), FM/TBM, TBM-hard, and TBM-easy categories. In template-based modeling, AlphaFold2 uses query–template sequence alignments to transfer structural information from known structures. Standard tools such as HHsearch produce these alignments using sequence similarity, but alignment quality degrades for remote homologs in the twilight zone. We hypothesized that OTalign's PLM-based realignment could rescue structural signal from poorly aligned templates.

We searched templates with HHsearch against PDB70, filtering to structures released before 2022-01-01 to prevent data leakage from CASP15 answer structures. For each of the 17 domains, we compared two single-sequence prediction conditions: HHalign templates with original alignments, and the same templates after OTalign realignment using Ankh-Large embeddings. OTalign additionally resorts templates by normalized PLM-based alignment score, which changes which four templates enter ColabFold's top-4 selection. All predictions used ColabFold v1.6.0 with AlphaFold2-ptm (5 models, 3 recycles). We evaluated TM-scores via USalign [Zhang et al., 2022], normalized by reference structure length.

S12.2 Per-Category Analysis

For TBM-hard domains, where templates exist but share limited sequence identity with the query, the mean TM-score improved from 0.728 to 0.764 (+0.035). The most striking case was T1176-D9 (TBM-hard), where OTalign realignment yielded a TM-score gain of +0.121 (0.630→0.751). Inspection of the template reranking revealed that OTalign promoted 6XJ9_B—a closer structural homolog—to rank 1 and replaced a poorly informative template (4O4A_A) with 6R3M_A, demonstrating that PLM-based alignment scores can identify better structural homologs that sequence-based ranking misses.

Similarly, for T1187-D1 (FM-classified but containing partial structural homologs), OTalign reordering of the top-4 templates improved the oracle-best TM-score by +0.066 (0.418→0.484; Table S7), although this gain did not transfer to pLDDT-best model selection. This highlights that *template ranking*—not just template availability—is a critical determinant of prediction quality, and that practical model selection can mask underlying improvements in structural diversity among sampled models.

For TBM-easy domains, OTalign provided a mean improvement of +0.012 (0.691→0.703), consistent with the expectation that even well-matched templates benefit from alignment refinement. In contrast, for FM domains lacking close structural homologs, neither HHalign nor OTalign templates provided meaningful structural signal (mean TM $\approx$ 0.42), confirming that template realignment cannot substitute for the absence of evolutionary information.

S12.3 Biological Implications

The mechanism behind OTalign's improvement is twofold: (1) OTalign produces more accurate query–template alignments that better position structurally equivalent residues, and (2) the PLM-based alignment scores provide a more biologically meaningful ranking of template quality, promoting structurally closer homologs into the top-4 positions used by AlphaFold 2. The category-dependent pattern of improvement—strongest for TBM-hard, moderate for TBM-easy, absent for FM—is consistent with our framework: OTalign enhances the *utilization* of existing structural information rather than creating signal where none exists. Notably, the gains concentrate on TBM-hard and FM/TBM domains—precisely the regime where templates exist but sequence-

based alignments are unreliable, so that improved alignment directly rescues structural signal. By contrast, TBM-easy targets are well served by any reasonable aligner, while FM targets lack informative templates altogether, rendering alignment quality moot; OTalign's benefit thus maps cleanly onto the region where template utilization is the bottleneck.

References

V. Boltyanski, H. Martini, and V. Soltan. *Geometric Methods and Optimization Problems*. Combinatorial Optimization. Springer US, 2013. ISBN 9781461553199.

J. Feydy, T. Séjourné, F.-X. Vialard, S.-i. Amari, A. Trouve, and G. Peyré. Interpolating between optimal transport and mmd using sinkhorn divergences. In K. Chaudhuri and M. Sugiyama, editors, *Proceedings of the Twenty-Second International Conference on Artificial Intelligence and Statistics*, volume 89 of *Proceedings of Machine Learning Research*, pages 2681–2690. PMLR, 2019. URL https://proceedings.mlr.press/v89/feydy19a.html.

T. Hamamsy, J. T. Morton, R. Blackwell, D. Berenberg, N. Carriero, V. Gligorijevic, C. E. M. Strauss, J. K. Leman, K. Cho, and R. Bonneau. Protein remote homology detection and structural alignment using deep learning. *Nature Biotechnology*, 42(6):975–985, 2023. ISSN 1546-1696. doi: 10.1038/s41587-023-01917-2.

K. Kaminski, J. Ludwiczak, K. Pawlicki, V. Alva, and S. Dunin-Horkawicz. plm-blast: distant homology detection based on direct comparison of sequence representations from protein language models. *Bioinformatics*, 39(10):btad579, 2023. ISSN 1367-4811. doi: 10.1093/bioinformatics/btad579.

W. Liu, Z. Wang, R. You, C. Xie, H. Wei, Y. Xiong, J. Yang, and S. Zhu. Plmsearch: Protein language model powers accurate and fast sequence search for remote homology. *Nature Communications*, 15(1):2775, 2024. ISSN 2041-1723. doi: 10.1038/s41467-024-46808-5.

F. Llinares-López, Q. Berthet, M. Blondel, O. Teboul, and J.-P. Vert. Deep embedding and alignment of protein sequences. *Nature Methods*, 20(1):104–111, 2022. ISSN 1548-7105. doi: 10.1038/s41592-022-01700-2.

M. Mirdita, K. Schütze, Y. Moriwaki, L. Heo, S. Ovchinnikov, and M. Steinegger. Colabfold: making protein folding accessible to all. *Nature Methods*, 19(6):679–682, 2022. ISSN 1548-7105. doi: 10.1038/s41592-022-01488-1. URL https://doi.org/10.1038/s41592-022-01488-1.

C. Orengo, A. Michie, S. Jones, D. Jones, M. Swindells, and J. Thornton. Cath—a hierarchic classification of protein domain structures. *Structure*, 5(8):1093–1109, Aug. 1997. ISSN 0969-2126. doi: 10.1016/s0969-2126(97)00260-8.

L. Pantolini, G. Studer, J. Pereira, J. Durairaj, G. Tauriello, and T. Schwede. Embedding-based alignment: combining protein language models with dynamic programming alignment to detect structural similarities in the twilight-zone. *Bioinformatics*, 40(1):btad786, January 2024. doi: 10.1093/bioinformatics/btad786.

M. Remmert, A. Biegert, A. Hauser, and J. Söding. Hhblits: lightning-fast iterative protein sequence searching by hmm-hmm alignment. *Nature Methods*, 9(2):173–175, 2012. ISSN 1548-7105. doi: 10.1038/nmeth.1818.

J. Söding. Protein homology detection by hmm-hmm comparison. *Bioinformatics*, 21(7):951–960, 2004. ISSN 1367-4803. doi: 10.1093/bioinformatics/bti125.

M. Steinegger and J. Söding. Mmseqs2 enables sensitive protein sequence searching for the analysis of massive data sets. *Nature Biotechnology*, 35(11):1026–1028, Oct. 2017. ISSN 1546-1696. doi: 10.1038/nbt.3988. URL http://dx.doi.org/10.1038/nbt.3988.

M. Steinegger, M. Meier, M. Mirdita, H. Vöhringer, S. J. Haunsberger, and J. Söding. HH-suite3 for fast remote homology detection and deep protein annotation. *BMC Bioinformatics*, 20(1): 473, 2019. ISSN 1471-2105. doi: 10.1186/s12859-019-3019-7.

T. Séjourné, G. Peyré, and F.-X. Vialard. *Unbalanced Optimal Transport, from theory to numerics*, pages 407–471. Elsevier, 2023. ISBN 9780323850605. doi: 10.1016/bs.hna.2022.11.003.

C. Zhang, M. Shine, A. M. Pyle, and Y. Zhang. Us-align: universal structure alignments of proteins, nucleic acids, and macromolecular complexes. *Nature Methods*, 19(11):1109–1115, 2022. ISSN 1548-7105. doi: 10.1038/s41592-022-01585-1.
